# A Phytoanticipin Derivative, Sodium Houttuyfonate, Induces *in Vitro* Synergistic Effects with Levofloxacin against Biofilm Formation by *Pseudomonas aeruginosa*

**DOI:** 10.3390/molecules170911242

**Published:** 2012-09-20

**Authors:** Jing Shao, Huijuan Cheng, Changzhong Wang, Yan Wang

**Affiliations:** Laboratory of Microbiology and Immunology, School of Chinese and Western Integrative Medicine, Anhui University of Traditional Chinese Medicine, Hefei 230038, China; Email: ustcsj@126.com (J.S.); ahwcz63@sina.com (C.W.); abudao2002@163.com (Y.W.)

**Keywords:** biofilm, sodium houttuyfonate, levofloxacin, phytoanticipin, *Pseudomonas aeruginosa*

## Abstract

Antibiotic resistance has become the main deadly factor in infections, as bacteria can protect themselves by hiding in a self-constructed biofilm. Consequently, more attention is being paid to the search for “non-antibiotic drugs” to solve this problem. Phytoanticipins, the natural antibiotics from plants, could be a suitable alternative, but few works on this aspect have been reported. In this study, a preliminary study on the synergy between sodium houttuyfonate (SH) and levofloxacin (LFX) against the biofilm formation of *Pseudomonas aeruginosa* was performed. The minimal inhibitory concentrations (MIC) of LFX and SH, anti-biofilm formation and synergistic effect on *Pseudomonas aeruginosa*, and quantification of alginate were determined by the microdilution method, crystal violet (CV) assay, checkerboard method, and hydroxybiphenyl colorimetry. The biofilm morphology of *Pseudomonas aeruginosa* was observed by fluorescence microscope and scanning electric microscope (SEM). The results showed that: (i) LFX and SH had an obvious synergistic effect against *Pseudomonas aeruginosa *with MIC values of 0.25 μg/mL and 128 μg/mL, respectively; (ii) ½ × MIC SH combined with 2 × MIC LFX could suppress the biofilm formation of *Pseudomonas aeruginosa *effectively, with up to 73% inhibition; (iii) the concentration of alginate decreased dramatically by a maximum of 92% after treatment with the combination of antibiotics; and (iv) more dead cells by fluorescence microscope and more removal of extracellular polymeric structure (EPS) by SEM were observed after the combined treatment of LFX and SH. Our experiments demonstrate the promising future of this potent antimicrobial agent against biofilm-associated infections.

## 1. Introduction

The biofilm theory was first proposed by Costerton [1] in 1978, and since then, people have realized that when bacteria are in the biofilm mode it makes them less sensitive to antibacterial agents, leading to high mortality due to repetitious infections [2]. Biofilmed bacteria are enveloped by polysaccharides, proteins, nuclear acids, *etc*. secreted by bacteria to protect them from antimicrobial agents; the biofilm is a self-defensive mechanism, and a structural and dynamical complex system [2]. *Pseudomonas aeruginosa* is one of the most widely-studied opportunistic human pathogens, and its biofilm is the main cause of many clinical infections, including chronic skin wounds [3], and the characteristic chronic lung infections of cystic fibrosis (CF) [4]. The biofilm formation tends to hinder enclosed bacterium from being killed, which is possibly related to different properties such as antimicrobial diffusion, physiological activity of inner and outer bacteria, quorum sensing, *etc.* [5]. 

To suppress the biofilm formation, many natural [6,7] and synthesized substances [8,9] have been explored. However, single antibiotic treatment is apt to induce more or less antibiotic tolerance, and cannot fully eradicate *Pseudomonas aeruginosa* biofilms. In terms of this problem, the combination of two antibiotics is emerging as an effective way to enhance the efficiency of biofilm inhibition and removal, and many antibiotics of microbial derivatives and metabolites have been applied in combination treatments [7,10].

Different from the traditional microbial antibiotics, the phytoanticipins [11], preexisting antimicrobial agents of medicinal plants that act against pathogen and insect invasions [12], present enormous potential for biofilm eradication and anti-pathogenic activity as a strong complement to microbial antibiotics [13]. As compared with antibiotics from microbes, the phytoanticipins have the advantages of lower toxicity, more broad-spectrum antibacterial activities, and more importantly, much lower bacterial resistance for a long-time dosage.

Houttuynin [*i.e.*, decanoyl acetaldehyde, CH_3_(CH_2_)_8_COCH_2_CHO], first isolated by Kosuge in 1952 [14], is one of the main and effective phytoanticipins extracted from *Houttuynia cordata* Thunb (Saururaceae family) with antimicrobial [15], antiviral [16], and anti-inflammatory activities [17]. Sodium houttuyfonate [SH, CH_3_(CH_2_)_8_COCH_2_CHOHSO_3_Na] is a derivative of houttuynin made by compounding with sodium bisulfite, soluble in hot water and alkaline solutions, but insoluble in chloroform and benzene, which exhibits broad-spectrum antibacterial activities, inhibits myocardial hypertrophy, prevents cardiac fibrosis, *etc.* [15,18]. It has been reported that SH and its homologues were more effective in suppressing Gram-positive (G^+^) bacteria, e.g., *Staphylococcus aureus* and *Bacillus subtilis*, than Gram-negative (G^−^) bacteria, e.g., *Escherichia coli* and *Pseudomonas aeruginosa* [19]. However, the limited reports were largely concerned with the inhibition of planktonic bacteria, and there have been no works reporting the control and removal of bacterial biofilms and the relevant encapsulated bacteria. 

In this work, we firstly used SH as a sole antimicrobial agent to investigate its suppression of *Pseudomonas aeruginosa* biofilm and alginate, one of the main component of the biofilm. Then, we employed SH as a synergistic agent for levofloxacin (LFX) to evaluate its anti-pathogenic activity on the formation of *Pseudomonas aeruginosa* biofilm.

## 2. Results and Discussion

### 2.1. MICs of LFX and SH on Pseudomonas aeruginosa

The minimal inhibitory concentrations (MICs) of SH and LFX were determined by the microdilution method, and they were 512 μg/mL and 2 μg/mL, respectively. According to the definition of fractional inhibitory concentration index (FICI) [20], the FICI of SH and LFX could be calculated by:


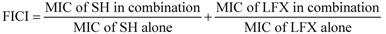
(1)

in which MICs of LFX and SH in combination were 0.25 μg/mL and 128 μg/mL, respectively. Thus, FICI was 0.375 here, *i.e*., less than 0.5, thus exhibiting a relative strong synergy of SH and LFX.

In our experiments, we begin to administrate in the medium after 24 h incubation. This medication mode mainly takes clinical applications into account. As biofilm forms, the infected focus will induce inflammation, and relevant medications commence. In other words, the biofilm may just be in the state of attachment (see Section 2.2) when the antimicrobial agents are applied.

### 2.2. Biofilm Suppression by SH Alone and in Combination with LFX

By numerous repeatable proteomic analyses, Sauer *et al*. put forward five stages of biofilm formation, namely: (i) reversible attachment (>0 min); (ii) irreversible attachment (2 h); (iii) maturation-1 (3 days); (iv) maturation-2 (6 days); and (v) dispersion (9–12 days) [21]. In terms of this rule, we chose 1 d, 3 d and 7 d as three time-points to observe the biofilm suppression and removal after medication. 

**Figure 1:**
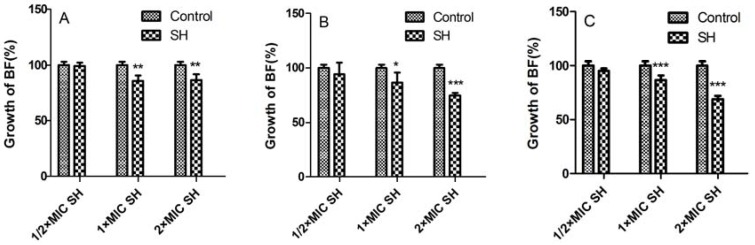
The effect of SH alone on the biofilm growth of *Pseudomonas aeruginosa*. *, *p *< 0.05; **, *p *< 0.01; ***, *p *= 0.000; n = 4. SH: sodium houttuyfonate.

Figure 1 shows control of SH alone on biofilm formation of *Pseudomonas aeruginosa*. In the 1st day (Figure 1A, ½ × MIC SH has nearly no effect on biofilm growth as compared with that in the control group, while 1 × MIC and 2 × MIC SH have obvious suppressive effects on biofilm (*p *< 0.01, n = 4). On the 3rd day (Figure 1B), there is no statistical significance of the impact by ½ × MIC SH treatment on biofilm, whereas, the growth of biofilm with 2 × MIC SH treatment (*p *= 0.000, n = 4) decreases even more than that with 1 × MIC SH treatment (*p *< 0.05, n = 4) as compared with the control group. In the 7th day (Figure 1C × MIC and 2 × MIC SH have exceedingly significant differences (*p *= 0.000, n = 4).

**Figure 2:**
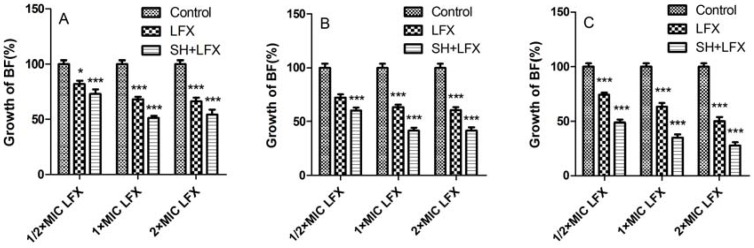
The synergistic effect of SH and LFX on the biofilm formation of *Pseudomonas aeruginosa*. *, *p *< 0.05; **, *p *< 0.01; ***, *p *= 0.000; n = 4. SH: sodium houttuyfonate; LFX: levofloxacin.

Figure 2 shows controls of LFX and SH + LFX on biofilm formation of *Pseudomonas aeruginosa*. In Figure 2A, LFX alone has obviously curbed biofilm formation, and except for the sample treated by ½ × MIC LFX (*p *< 0.05, n = 4), the differences in other two cases by 1 × MIC and 2 × MIC LFX are extremely significant (*p *= 0.000, n = 4) as compared with control. After combination with SH, the biofilm growths are all lower than the corresponding ones treated by LFX alone (*p *= 0.000, n = 4). Similar results also appear in Figure 2B (day 3). With the extension of biofilm development, the synergistic effects in Figure 2C (day 7) are even more notable.

From the above results, it could be observed that the effect of SH alone on biofilm control is limited, as even the maximum dosage of SH (=2 × MIC) can merely restrain about 30% biofilm growth (Figure 1C). This may be related to the fact that SH is more active on Gram-positive (G^+^) bacteria than Gram-negative (G^−^) ones [19]. However, the combination of SH with LFX exhibits strong (more than 50% biofilm growth in Figure 2C) and persistent (7 days) inhibition of biofilm. More importantly, SH increases the biofilm suppression in combination with LFX. That is to say, phytoanticipins may have a nice synergistic effect with microbial antibiotics. Next, it was of interest to probe into the synergistic effect of SH and LFX on alginate production.

### 2.3. Alginate Removal by SH Alone and in Combination with LFX

Alginate is an acetylated polymer, composed of non-repetitive monomers of β-1,4-linked L-guluronic and D-mannuronic acids [22]. Until now, it has been assumed to be one of the most critical factors contributing to antibiotic tolerance and immune evasion, and not just playing a structural role [23,24,25]. Recently, Mann and Wozniak published a comprehensive review on this aspect [26]. Thus, it is necessary to investigate the effect of SH on alginate inhibition in combination of LFX.

**Figure 3:**
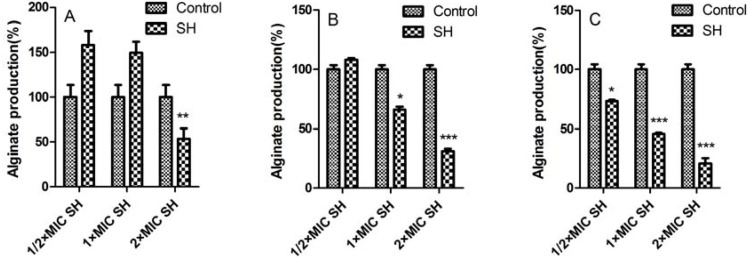
The effect of SH alone on alginate production of *Pseudomonas aeruginosa* biofilm. *, *p *< 0.05; **, *p *< 0.01; ***, *p *= 0.000; n = 4. SH: sodium houttuyfonate.

Figure 3 shows the effect of only SH on alginate production of *Pseudomonas aeruginosa* biofilm. It is interesting to observe that: (i) alginate is still produced on the first day after treatment of ½ × MIC SH and 1 × MIC SH, and its concentration decreased dramatically to about ⅓ (*p *< 0.01, n = 4) after increasing the dosage of SH to 2 × MIC (Figure 3A); (ii) after three-day medication, the alginate concentration is still a little higher than the control concentration when using ½ × MIC SH, but then becomes lower and has statistically significant differences (*p *< 0.05 and *p *= 0.000, n = 4) with the respective dosages of 1 × MIC SH and 2 × MIC SH (Figure 3B); and (iii) after administration until the seventh day, the inhibitions of alginate become more and more remarkable with the addition of SH from ½ × MIC to 2 × MIC, as the alginate productions are reducing continuously (*p *< 0.05, *p *= 0.000 and *p *= 0.000, n = 4, Figure 3C).

**Figure 4:**
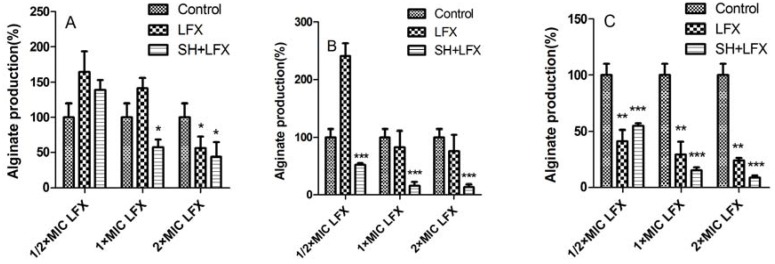
The synergistic effect of SH and LFX on the alginate production of *Pseudomonas aeruginosa* biofilm. *, *p *< 0.05; **, *p *< 0.01; ***, *p *= 0.000; n = 4. SH: sodium houttuyfonate; LFX: levofloxacin.

In Figure 4, the synergistic effect of SH and LFX is evaluated. When treated by LFX alone, the situations are similar to those in Figure 3. The suppressions of alginate production basically have no statistical significance (Figure 4A,B) as compared with those in control group, which is changed until biofilm is treated for seven days (*p *< 0.01, n = 4, Figure 4C × MIC and 2 × MIC SH in day 1 (*p *< 0.05, n = 4, Figure 4A), and the effects become even more prominent in day 3 in all three dosage (*p *= 0.000, n = 4, Figure 4B). The trend is kept to the day 7 (*p *= 0.000, n = 4, Figure 4C

Similar to the case in Figure 1, SH alone has a mild influence on alginate production (Figure 3). But with the addition of LFX, their synergistic effects gradually impose great limitations on alginate production (Figure 4). As known, alginate is secreted by bacteria to form an extracellular matrix, and finally encase the bacteria to help them hide from the toxic effects of antimicrobial agents. In Figure 2C and 4C the reduction of biofilm growth leads to the stagnancy or loss of alginate production inferring that alginate may at least partly participate in biofilm formation. 

### 2.4. In Vitro Antimicrobial Role of SH and Its Synergistic Activity with LFX

It can be observed that the growth of biofilm decreases from 99 ± 2.77% after one-day administration of ½ × MIC SH to 69 ± 3.07% after seven-day administration of 2 × MIC SH in Figure 1, and the alginate production lowers from 158 ± 15.9% after one-day medication to 21 ± 4.61% after seven-day medication in Figure 3. Thus, SH exhibits a certain antimicrobial activity *in vitro*.

It can be seen that the biofilm growth loses about 45% between one-day therapy of ½ × MIC SH + ½ × MIC LFX and seven-day therapy of ½ × MIC SH + 2 × MIC LFX in Figure 2, and the alginate production drops about 131% between one-day treatment of ½ × MIC SH + ½ × MIC LFX and seven-day treatment of ½ × MIC SH + 2 × MIC LFX in Figure 4. Therefore, SH has a very intense synergy with LFX in the restriction of biofilm growth and alginate production.

From Figure 1 to Figure 4, it can be manifested that: (i) both indexes, *i.e.*, growth of biofilm and alginate production, are dosage-dependent, because they display a decreasing tendency with the addition of LFX or SH; (ii) both items are time-dependent, as the suppression effects become better with the elongation of administration from day 1 to day 7. As for the synergistic effect of SH and LFX, it is also interesting to note that the alginate production effects are not synchronous with those of biofilm growth, which can be inferred from two aspects: (i) the alginate production still increases in comparison with control when employing a lower dosage of ½ × MIC LFX + ½ × MIC SH, which is more noteworthy in the first day of dosage (= 139 ± 13.52%, Figure 4A), suggesting no effective suppression of the combined antibiotics on alginate production; in the same period, the growth of biofilm is 73 ± 3.9% by SH in combination of LFX, showing a certain inhibition (Figure 2A); (ii) the alginate production is only 8 ± 1.71% when using a higher dosage of 2 × MIC LFX + ½ × MIC SH after seven days treatment (Figure 4C), while the growth of biofilm accounts for 28 ± 3.06% with the same time-span (Figure 2C), higher than that of alginate production. These discrepancies may be caused by following reasons: (i) in the stage of initial attachment (the first day), some planktonic bacteria are suppressed or killed by antibiotic alone or in combination, leading to biofilm reduction, while other irreversibly-attached bacteria begin to synthesize and secrete alginate in large amounts; (ii) the alginate is mainly produced by the above bacteria which have enough nutrients to continue their metabolism, but the inner bacteria tend to remain in a dormant mode due to limited nutrition, causing them to escape the destruction by combined antibiotics [5]. As a result, when the alginate production is restrained, the encased bacteria mentioned above are exposed to be killed, while the continuous killing of the inner bacteria still needs time and re-addition of antibiotics in combination; and (iii) we cannot rule out other factors, e.g., quorum sensing (QS), that might influence biofilm formation except the production of alginate, which is still under investigation in our lab.

### 2.5. Observation of Pseudomonas aeruginosa by Fluorescence Microscope

**Figure 5:**
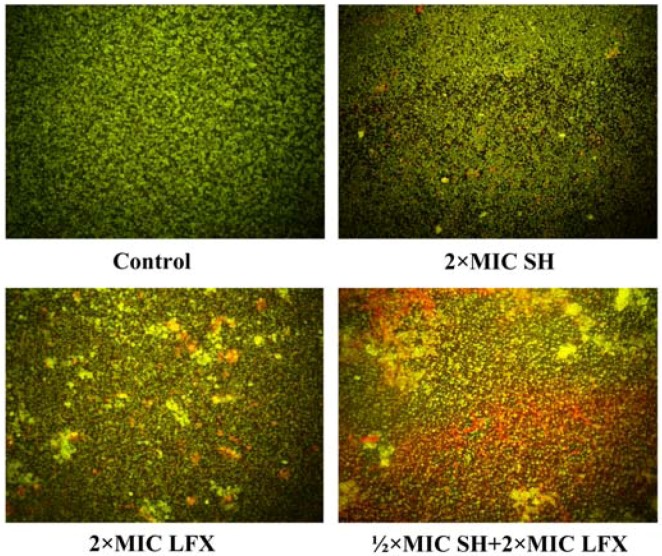
Observation of bacterial viability by BX51 fluorescence microscope (×400) under the treatment of no drugs, 2 × MIC SH, 2 × MIC LFX, and ½ × MIC SH + 2 × MIC LFX, and the exposure of 100 μg/mL FAD for 5 min and 60 μg/mL PI for 5 min. Green: living cells stained by FAD; Red: dead cells stained by PI. Other conditions are given in Section 3.7.

Figure 5 presents the fluorescence microscope images of the bacteria. The FAD-PI dyed bacteria are treated by SH alone, LFX alone, and SH + LFX. Living bacteria exhibit green and dead bacteria show red. 

In Figure 5, it can be observed that: (i) living bacteria are scattered in control group as all cells are seen to be green; (ii) most bacteria are alive and less are dead as most cells are recorded to be green with less red in 2 × MIC SH group and 2 × MIC LFX group, however, the bacteria viability in the 2 × MIC LFX group is notably less than that in 2 × MIC SH group which has more red cells in view; and (iii) less bacteria in the ½ × MIC SH + 2 × MIC LFX group are alive than those in the SH alone group and LFX alone group as more red dots appear in the image. From the four images, the quantities of dead cell exhibit an increasing tendency, indicating the antimicrobial effects promoted by SH and LFX in combination. 

### 2.6. Observation of *Pseudomonas aeruginosa* Biofilm Morphology by SEM

**Figure 6:**
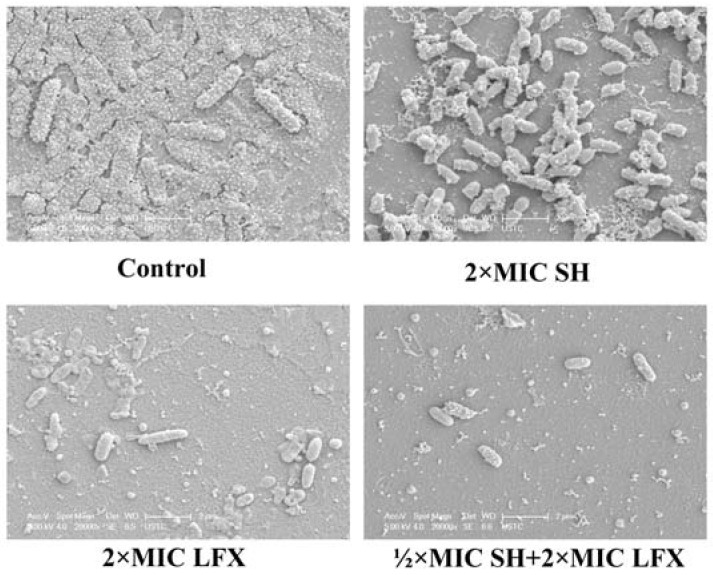
Observation of bacterial biofilm morphology by SEM.

Figure 6 shows is the morphology of the bacterial biofilm by SEM. In the control group, the bacteria are largely covered by mucous substances which are mainly composed of alginate, and dense cells are seen to exist in the whole view, which is similar to that in the control group in Figure 5. In the 2 × MIC SH group, the mucous substances are largely removed, but the bacteria are not completely inhibited, indicating that the SH is mainly suppressing the production of mucuous substances. In terms of the results that the alginate production (%) is about 21% (Figure 3C) after the treatment of 2 × MIC SH, it is obvious that the alginate is the main substance in the mucuous substances and SH largely targets the alginate. In the 2 × MIC LFX group, both mucous substance and bacteria are dramatically decreased, however, there are still some matrix substances left, revealing the relative strong antipathogenic of LFX on alginate and encased bacteria. In the ½ × MIC SH + 2 × MIC LFX group, both mucous substance and bacteria are wiped out further compared with those in the 2 × MIC LFX group, reflecting that SH can enhance the suppressive effects of LFX on alginate to kill the bacteria under the mucus substances, *i.e.*, SH and LFX have better synergistic effects on biofilm removal.

## 3. Experimental

### 3.1. Strains and Materials

**Figure 7:**
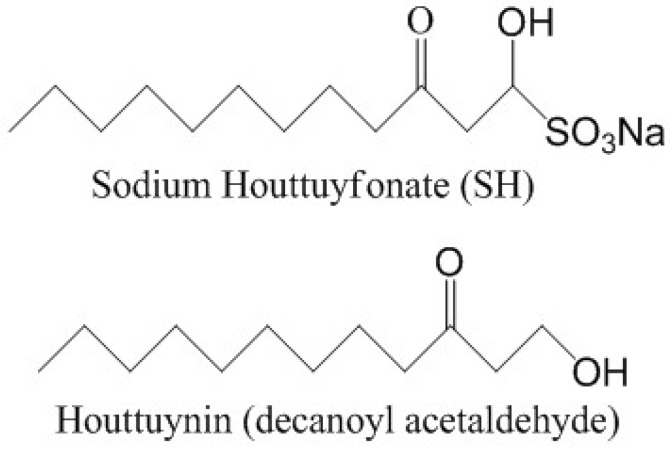
The chemical structures of sodium houttuyfonate (SH) and houttuynin (decanoyl acetaldehyde).

*Pseudomonas aeruginosa* ATCC27853, sodium houttuyfonate (SH, Figure 7) and levofloxacin (LFX) were all obtained from the National Institute for the Control of Pharmaceutical and Biological Products (NICPBP, Beijing, China). The LB, MH and TSB media were purchased from Aoboxing Bio-tech Co., Ltd. (Beijing, China). Crystal violet solution was purchased from bioMérieux (Lyon, France). The alginate standards, fluorescein di-*o*-acetate (FDA) and propidium iodide (PI) were obtained from Sigma (St. Louis, MO, USA).

### 3.2. Cultivation of Pseudomonas aeruginosa

*Pseudomonas aeruginosa* ATCC27853 was inoculated in LB broth, cultivated in a constant-temperature shaker GLY (Fuma, Shanghai, China) of 220 rpm for 6 h at constant 37 °C. Then the bacteria were harvested by GL-20G-Ⅱ high-speed refrigerated centrifuge (Fuma) at 2,000 r/min for 10 min. The supernate was discarded, and the precipitate was resuspended with pH 7.2 phosphate-buffered saline (PBS), centrifuged again at 2,000 r/min for 10 min. The collection was mixed with PBS pH 7.2, and adjusted to 2 × 10^5^ CFU/mL by the microdilution method. 

### 3.3. Preparation of SH and LFX Solutions

Both stock solutions of SH and LFX were prepared freshly in MH broth and filtered through a 0.22 µm Millipore filter (Millipore, Billerica, MA, USA), and their concentrations were adjusted to 160 and 32 µg/mL, respectively. In the MIC determination experiments, SH was twofold-diluted into ten concentration gradients by MH medium at a final concentration from 0.03125 to 16 mg/mL, and LFX was diluted to a final concentration ranging from 0.0625 to 32 µg/mL.

### 3.4. MIC Measurement

Both SH and LFX were added into 96-well plates with eight wells in duplicate for each concentration gradient, and each well had both 100 μL either SH or LFX solution and 100 μL inoculum with the final concentration of 1 × 10^5^ CFU/mL. The plates were incubated for 24 h at 37 °C, and then the bacteria were inoculated on nutrient agar medium and incubated for another 24 h at 37 °C. The MIC was determined as the minimum drug dilution with no growth of bacteria on plates. Each MIC assay was performed in triplicate. The checkerboard microdilution method was adopted for antimicrobial assay of SH and LFX in synergy. The concentrations of combined antibiotics were adjusted from ⅛ × MIC to 2 × MIC, and 100 μL drugs were added into each well. A volume of 100 μL/well inoculum was mixed with the drugs to the final concentration of 1 × 10^5^ CFU/mL. Then, the setup was incubated at 37 °C for 24 h to observe the growth of bacteria, which was detected by a 318-microplate reader (Sanco Instruments, Shanghai, China). The control had medium and inoculum without any antimicrobial agents. Each test was performed in triplicate [27].

### 3.5. Effects of SH Alone and SH + LFX in Combination on Anti-Biofilm Formation

Ten groups were tested: control, ½ × MIC SH, 1 × MIC SH, 2 × MIC SH, ½ × MIC LFX, 1 × MIC LFX, 2 × MIC LFX, ½ × MIC SH + ½ × MIC LFX, ½ × MIC SH + 1 × MIC SH, ½ × MIC SH + 2 × MIC LFX (Figure 1 and Figure 2). In a 96-well plate, each was added with 200 μL *Pseudomonas aeruginosa* suspension, incubated at 37 °C with no shaking. After 24 h, the strain suspension was discarded, and the planktonic microbes were washed by PBS, inoculated into TSB medium with the above concentrations of antibiotics. The control was cultivated in the medium with no antibiotics. According to the description of O’Toole [28], the medium were exchanged for a new one with antibiotics every other day. At the end of the 1st, 3rd and 7th day, 4 °C PBS was employed to wash the planktonic bacteria on the 96-well plate. Then, each well was added into 200 μL 1% crystal violet solution for 20 min. After they were rinsed with de-ionized water till no crystal violet was visible, the wells were dried and 95% alcohol added to destain. The destained samples at the end of 1st, 3rd and 7th day of medications were transferred to a cuvette and diluted with 95% alcohol (3 mL) to determine the OD value (OD_b-sample_) at the wavelength of 570 nm. The corresponding OD values of negative control (OD_b-control_), consisting of biofilm without any medications, were treated as the above steps and set as 100% at the end of 1st, 3rd and 7th day of medications. Then, the growth of biofilm (%) could be calculated for each group as follows:


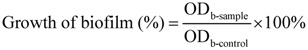
(2)

and each calculation was processed in triplicate.

### 3.6. Effects of SH Alone and SH + LFX in Combination on Anti-Alginate Production

There were also ten groups, the same as those in Section 3.5. The sterile cover-glass carrier was put into the 6-well culture plate, and then TSB medium (2 mL) and strain suspension (0.2 mL) were added. After incubating for 1 day at 37 °C, the medium was discarded, and the cover-glass was taken out to wash the planktonic bacteria out with sterile PBS. The cover-glass was then put into the well again. The antibiotic groups were given medium containing antibiotics, while the negative control used medium without antibiotics. All the above manipulations were repeated in each 24 h. At the end of the 1st, 3rd and 7th day of medication, the cover-glasses were fetched out to rinse the planktonic bacteria by PBS. Then, the cover-glasses were put into test tube with additions of PBS (6 mL), sulphuric acid and sodium borate (1.2 mL each), boiled for 5 min, and placed at 4 °C. 1% Hydroxybiphenyl (20 μL) was added to the glass for colorization, and vibrated for 30 min by ultrasound. The OD values at the end of 1st, 3rd and 7th day of medications were recorded at the wavelength of 570 nm (OD_a-sample_). According to the above treatments, the corresponding OD values of negative control (OD_a-control_), consisting of alginate without any medications, were set as 100%, and then the alginate production (%) could be calculated for each group as follows:


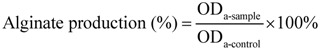
(3)

and each calculation was conducted in triplicate.

### 3.7. Fluorescence Microscopy of Bacteria

The carrier disposition was similar to that described in Section 3.6. After the glass was removed and the planktonic bacteria rinsed with PBS at the end of the 7th day of medication, it was stained with fluorescein diacetate-propidium iodide (FDA-PI) fluorescent dyes to observe the vitality of dispersed bacteria, in which FDA concentration was 100 µg/mL, and PI concentration was 60 µg/mL. The bacteria were first stained by FDA for 5 min, and then with PI for 5 min. The dead bacteria were stained as red by PI, while the alive ones were as green by FDA. A BX51 fluorescence microscope was used (Olympus, Tokyo, Japan) to record the results.

### 3.8. Scanning Electric Microscope of Biofilm Morphology

The biofilm morphology was observed by silver-staining on a SEM (Sirion200, FEI, Columbia, MD, USA).

## 4. Conclusions

A phytoanticipin derivative, sodium houttuyfonate, was adopted to investigate its synergistic effects with levofloxacin on the suppression of *Pseudomonas aeruginosa* biofilm. We separately evaluated the inhibitions by SH alone and LFX alone on biofilm formation and alginate production, and the synergistic effect of SH with LFX on biofilm formation and alginate production. The results demonstrate that SH has robust synergistic activity with LFX, and can dramatically decrease the amount of bacteria by effective inhibition of biofilm formation and alginate production. Consequently, phytoanticipins represent a promising alternative to coordinate with microbial antibiotics, playing an even greater role in anti-biofilm treatment.
